# Evaluation of the Char Formation During the Hydrothermal Treatment of Wooden Balls

**DOI:** 10.1002/gch2.202300169

**Published:** 2023-11-05

**Authors:** Jens Pfersich, Pablo J. Arauzo, Pierpaolo Modugno, Maria‐Magdalene Titirici, Andrea Kruse

**Affiliations:** ^1^ Conversion Technologies of Biobased Resources University of Hohenheim Garbenstrasse 9 70599 Stuttgart Germany; ^2^ School of Engineering and Materials Science Queen Mary University of London Mile End Road London E1 4NS UK; ^3^ Department of Chemical Engineering Imperial College London South Kensington London SW7 2AZ UK

**Keywords:** HTC, hydrochar, primary char, pyrochar, pyrolysis, secondary char, wood

## Abstract

With wooden balls, a visualization of the hydrothermal carbonization to show the progress of the conversion to char is presented. In the present study, the balls represent the particles of biomass to investigate the differences in conversion outside and inside of biomass particles, during hydrothermal carbonization. A special focus is on hydrochar and pyrochar formation. The wooden balls are treated in subcritical water at 220 °C for holding times between 0 and 960 min. Even after 960 min, hydrolysis of the original biomass is incomplete as cellulose and hemicellulose are linked by lignin, inhibiting the reaction with water. Moreover, two different pathways of char production can be observed. Inside of the wooden ball pyrochar is formed as any water got that deep in, on the surface hydrochar is fixed, originated from the surrounding liquid. On the ground of the HTC reactor, a thin, brittle precipitate of likely hydrochar or humins can be found either from the precipitation of loosely attached compounds on the surface of the biomass or direct precipitation from the liquid.

## Graphical abstract

Wooden balls are treated with subcritical water under hydrothermal conditions to produce platform chemicals, hydrochar, and pyrochar. The conversion of lignocellulosic material starts at two areas, the surface of the wooden balls as well as in the center with a transition layer, which size decreases with increasing holding time, in between.

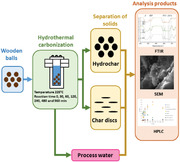

## 1 Introduction

As part of an evolving world, sustainability becomes more and more important in an industry and society of growing needs. Working with sustainable natural resources is already part of several production processes, where usually dry biomass is of major importance.^[^ ^1^, ^2^ ^]^ Nowadays, the wet biomass is mainly used for biogas production. Nonetheless, for energy generation by solid fuel production, the wet biomasses are interesting, however still unused, raw materials, which are suitable for hydrothermal carbonization (HTC).^[^ ^3^ ^]^ Moreover, no drying of the raw materials is needed for biomass, as HTC works with water as medium and reagent. Thus, the main disadvantage for the application of dry charring processes like pyrolysis, drying of wet biomass, is not relevant for HTC.^[^ ^4^, ^5^ ^]^ Since the energy supply to reach the relative low reaction temperatures is the only other necessity for HTC besides water and biomass, it is a sustainable and easy applicable process to produce a variety of products. These products are not only solid fuels but also carbon materials for different applications.^[^ ^6^ ^]^

Generally, HTC is a thermochemical process, which takes place in a temperature range between 180 to 260 °C under autogenous pressure of around 24 MPa.^[^ ^7^, ^8^, ^9^ ^]^ These operating conditions enhance the decomposition of the chemical compounds and the production of platform chemicals like 5‐hydroxymethylfurfural (HMF), acids like lactic acid and chars.^[^ ^8^, ^10^ ^]^ There are two different types of chars discussed in literature – primary and secondary char. Primary char, which is referred to as pyrochar, which resembles the result of the solid‐to‐solid conversion mechanisms of mainly nondegradable biomass compounds under HTC conditions such as lignin.^[^ ^11^, ^12^ ^]^ The secondary char is also related to as hydrochar due to the polycondensation reactions it is formed by.^[^ ^11^, ^12^ ^]^ Their similar higher heating value and carbon content compared to lignite turn them into good alternatives for fossil fuels.^[^ ^6^, ^13^ ^]^

More in detail, the conversion of the biomass during the HTC process is a consequence of several reaction pathways according to literature.^[^ ^5^, ^6^, ^14^ ^]^ The first is the hydrolysis of the lignocellulosic compounds. These are hemicellulose, cellulose and lignin if a lignocellulosic biomass like beech wood is used.^[^ ^15^, ^16^, ^17^, ^18^ ^]^ Monomers of hemicellulose and cellulose are sugars, which are easily soluble in water. Thus, the second important effect of water besides its use as reaction medium, is the use as reaction promoter: In the liquid phase, the tautomerism of hexoses (C6) and pentoses (C5) occurs and leads to the formation of HMF by dehydration, consecutively.^[^ ^19^, ^20^ ^]^ HMF is further converted to form hydrochar,^[^ ^21^, ^22^, ^23^ ^]^ which might surround pyrochar in a spherical appearance.^[^ ^24^, ^25^ ^]^

A deeper understanding of the mechanisms of HTC is the general interest, especially considering the possible scale‐up.^[^ ^26^, ^27^ ^]^ Optimization of the HTC process is accomplished by understanding the mechanisms that result into each desired product. A fundamental understanding of char production is important because of the possible applications as carbon electrodes, activated carbon and simple, solid fuel.^[^ ^28^, ^29^, ^30^ ^]^ Especially advanced carbon materials like carbon electrodes, tailor‐made by adjusting the production conditions, is the goal. Attempts were made to investigate the reaction kinetics in literature before, but the mechanism of the solid‐to‐solid conversion is still unclear.^[^ ^19^, ^22^, ^31^, ^32^ ^]^ As proposed earlier in previous studies, the likeliest mechanism is pyrolysis, more precise torrefaction, taking place in the inner layers of biomass particles.^[^ ^11^, ^12^ ^]^ In contrast to torrefaction, HTC needs less heating energy because the water is part of the reaction process, which has consequences on thermodynamics and kinetics. Compared to “dry” pyrolysis. the presence of water opens a completely different reaction pathway.

This gets clearer considering the reaction enthalpies of wood conversion. The reaction enthalpy for the HTC of wood is reported in literature as −2.5 to −3.5 MJ kg^−1^, while the pyrolysis is less exothermic with *H*_R_ = −0.7 MJ kg^−1^.^[^ ^33^, ^34^ ^]^

**Figure 1:**
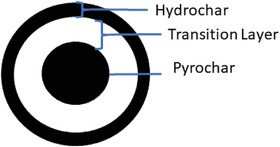
Scheme of the assumption for the behavior of wooden balls during HTC.

Moreover, in “dry experiments” there is a difference between the outer and the inner material of a particle. Some studies reported a thin reactive layer caused by pyrolysis covering the raw biomass core, which was caused by the different temperatures at the surface and inside of the particles.^[^ ^35^, ^36^ ^]^ However, for HTC the appearance between the surface and the core of the particles should be slightly different due to the presence of water (**Figure** **1**).

In addition, a core–shell structure of solid particles produced from glucose was already presented by Higgins et al.^[^ ^37^ ^]^ While glucose is a simple model molecule, the question if the same behavior could be seen in a real biomass, should be answered as the next step to understanding HTC. Furthermore, information on how this effect can be controlled to produce only the desired product should be gathered. These information are vital to producing platform molecules, tailor‐made chars and in the same time separating phosphates of real biomasses. In an attempt to include the vast majority of biomasses, a complex biomass with the most complex and abundant bio macromolecules should be used. As wood is made of bio macromolecules like cellulose, hemicellulose and lignin together with many other smaller molecules, wood is an interesting biomass to use. Plus, the solid product keeps the shape of the original biomass, which helps to evaluate the structure inside to follow the steps of char production and distinguish hydrochar from pyrochar.

The purpose of this study is the evaluation of the effect of an increasing retention time on a low ash containing biomass (wood) to give a better assessment of the effects of HTC compared to “dry” pyrolysis and in competition with the pyrolysis pathway. Here, balls with a diameter of 12 mm made from beech wood are used to be able to see different layers throughout several retention times and still have a good heat transfer within the biomass. The hypothesis is, that, according to Figure 1, with increasing retention time, the hydrochar layer on the surface of the ball grows larger as well as the pyrolysis of the core of the wooden ball (WB). After some time, the transition layer of (nearly) unconverted biomass dissipates.

## 2 Results

### 2.1 Moisture Content

Fresh cut wood exhibits a high moisture content (around 60%, depending on the part of the tree taken).^[^ ^38^ ^]^ Consequently, to be used in industrial processes, wood needs to be dried first to decrease the moisture content below 30%, which is the fiber saturation point, below which shrinking of the wood starts.^[^ ^39^, ^40^ ^]^ The water content of the wood used in these experiments was determined to be (8.96 ± 0.05) %.

Water is mainly retained in the capillaries of the lignocellulosic fibers and between those. The fibers of the WBs are bound together by lignin, which is the main reason that hinders the access of water to the fibers.^[^ ^41^ ^]^ Thus, swelling of the wood takes longer than swelling a model compound like pure cellulose, in contact with the swelling medium water.

Previous to the HTC experiments, the swelling of the WBs in water was examined. As they were stored in a sealed package, the same starting criteria for all the WBs were assumed. The uptake of water by the WBs is listed in the Supporting Information (Table S1, Supporting Information).

By using the same experimental setup like the one used during the HTC experiments (reactor filled with six WBs and the same amount of water), the total uptake of water and therefore the degree of swelling was calculated. By dividing the mass of the WB after swelling by the original mass of the WB, multiplied by 100% and subtracting 100% from the result, the percentage of swelling equals the percentage of water taken up referring to the mass of the original biomass. After one hour, the swelling reached a notable degree by increasing the mass of the WBs by more than 10 wt% This meant that, as soon as the reaction temperature reached (retention time 0 min) the WBs increased their mass by at least 10 wt% with the uptake of water. However, this does not reveal anything about how deep the water penetrated the WBs.

In the experiments, the WBs swelled to a certain degree. In the areas, in which the water can penetrate, this has two effects: First, a better heat transfer, and second, reactions with water may occur. To ensure constant conditions during the HTC, the balls were left in deionized water for 30 min to swell before starting the HTC treatment. Based on the swelling experiments, the water content and gradient in the WBs is assumed to be similar. On the one hand, the possibility of water supported reactions should be similar for all WBs in the batch. On the other hand, as the structure of wood is caused by its fibers, the swelling of each WB is unequal for every direction. Furthermore, it is assumed that the water penetrates equally in the WBs during the experiments.

**Figure 2:**
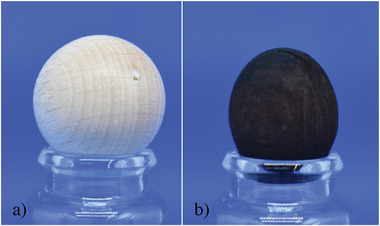
Comparison of two wooden balls, one before HTC a) and one after 30 min b).

**Figure 3:**
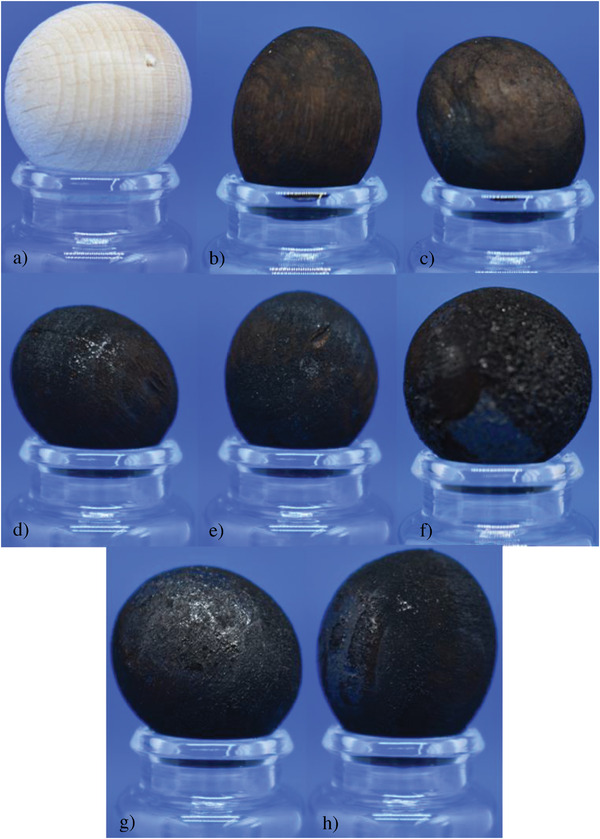
Comparison of the size and form of the WB, as well as the degree of carbonization: a) raw, b) 0 min, c) 30 min, d) 60 min, e)120 min, f) 240 min, g) 480 min and h) 960 min.

During HTC the shape of the WBs changes from spherical to oval (**Figures** **2** and **3**). HTC seems to be more distinct in the direction of the fibers, compared to the direction across the fibers, and therefore creates the oval form, which stays constant even after drying. This is supported by the unequal character caused by the fibers as water penetrates between them but not into the fibers.

### 2.2 Visual Analysis

#### 2.2.1 Appearance of the Balls

The visual comparison between the initial wooden ball and the ball after HTC for 30 min (Figure 2) was done to get a better understanding of the effect of the HTC parameters (e.g., pressure and retention time) onto the structure of the WB.

First, the outer shell of the WBs after HTC (Figure 2b) shrank compared with the original one (Figure 2a). The WB before the HTC is light brown and spherical shaped, while the ball on the right side, the one after the HTC, is darker brown or even black and more oval.

In Figure 3b–h the changes in the outer appearance of the converted balls during the retention times, which is always different compared with the original one, are shown. Moreover, different shades of black and differences in the appearance (glossiness, hapticity, etc.) make each process unique.

**Figure 4:**
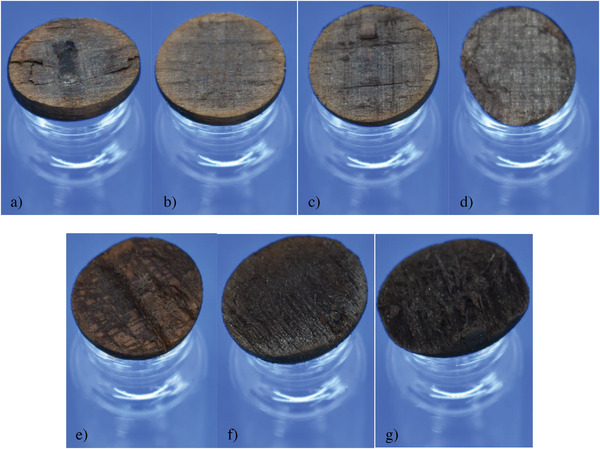
Wooden ball converted at 220°C for a) 0 min, b) 30 min, c) 60 min, d) 120 min, e) 240 min, f) 480 min and g) 960 min: For shorter retention times, brighter area below the surface which turns as dark as the core over retention time.

After focusing on the outer appearance of the WBs, changes of the inner of the WBs to find out more about the different layers as projected in Figure 1, are the next step. **Figure** **4** shows half‐cut converted WBs after all retention times (30 min to 960) to find out more about the effects of the retention time on the changes mentioned before.

Digging deeper into the balls, the color changes again in shades. While the outer shell is completely black, the area underneath is brighter for short retention times than for longer retention times. Furthermore, in the beginning two clearly separated black areas can be determined as well as a brighter layer in between. This is in good agreement with Figure 1, where this appearance was assumed. By increasing retention time, this brighter layer disappears while the black of the core seems to grow larger.

With longer retention time, the texture of the balls became foamy with the least stable structure for the longest retention time. While the outer shell still is brittle, the remaining part of the balls get decomposed into small pieces and fibers by applying small forces like scratching the outer layer.

**Figure 5:**
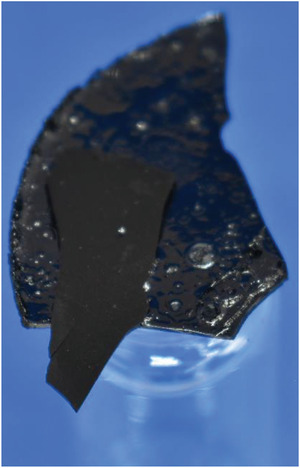
Brownish D after 960 min at the bottom of the reactor with one pale and one glossy side looking like (molten) plastics.

In addition to the color modification of the WB after HTC, two clear effects are observed. I) The black “shell” of the balls, that appeared after 30 min, could be washed off with hot water. II) After 60 min (**Figure** **5**), a brownish solid appeared at the bottom of the reactor and turned darker the longer the retention time was chosen.

The brownish precipitate that appeared at the bottom of the reactor after HTC at 220 °C for 960 min is shown in Figure 5. Due to its appearance as one solid part covering the entire ground of the reactor, as well as its smooth surface, the brownish solid is referred as disk (D).

This D was attached to the bottom of the reactor, hence a spatula was needed to remove it, which is the reason why all of the discs broke into parts as they are brittle. The upper side of the D is glossy and raw while the side attached to the bottom of the reactor is pale and smooth. Moreover, the thickness of the D increases with increasing retention time.

From here on, the hydrothermal treated balls as well as the material obtained thereof were labelled as S‐TTT‐XX, where S is the residual solid component, either WB or D, TTT is the retention time of the HTC and XX is W (washed material) or NW (nonwashed material).

#### 2.2.2 Scanning Electron Microscopy (SEM)

**Figure 6:**
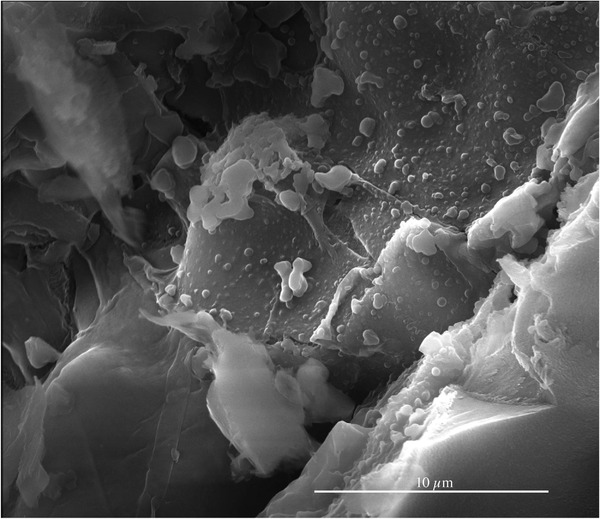
SEM image (HV 20.00 kV, mag 10 000× and WD 11.4 mm) of the outer layer of the wooden ball converted at 220 °C for 0 min. Some spherical particles are attached to the biomass or char surface.

The surface structure of the surface of a hydrothermal treated WB (0 min retention time) and the resulting chars are shown in **Figure** **6**. Here, the initial structure of the WB seems to have changed under the influence of the thermal treatment. The discussion of the different appearances of the WBs before and after the HTC already indicates that there are structural differences in the balls.

Compared to the raw biomass, which consists of smooth fibers and areas of wood, the sample which was cooled down directly after reaching the reaction temperature (0 min) shows first signs of conversion (Figure 6). Spherical particles appeared on the fibers as well as larger areas with a different texture than the raw biomass.

**Figure 7:**
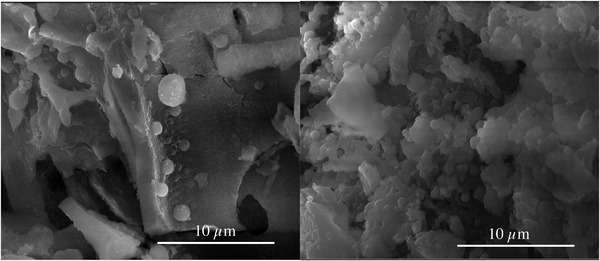
SEM images (HV 20.00 kV, mag 10 000× and WD 11.4 mm) of two samples of the outer layer of the WB after 480 min of HTC to compare the surface of a washed (left) and not washed (right) sample.

To further evaluate the surface, the hydrothermal treated WBs were washed with demineralized water after separation of the liquids. Depending on the changes of the surface before and after washing, the char and its link to the surface can be discussed. For this issue, **Figure** **7** shows the comparison of a WB‐480‐W and WB‐480‐NW.

The WB‐480‐NW shows a lot of medium sized spherical particles, mostly joint or molten together while the WB‐480‐W shows a few large, some mediocre sized and a lot of very small spherical particles.

### 2.3 Physicochemical Properties of the Produced Hydrochars

#### 2.3.1 Yield and Composition of Hydrochar

The yield of hydrocar is listed in the supporting information in Table S2 (Supporting Information). The increase of the retention time results in a decrease of the yield of hydrochar (wt%) of the WB‐W and WB‐NW.

As expected, the yield of hydrochar decreases with increasing retention time, from 64 wt% (0 min) and at around 40 wt% (480 min). While for short retention times literature also reports about hydrochar yields of around 65 wt%, the yield with increasing retention time decreases to a smaller extent.^[^ ^42^, ^43^, ^44^ ^]^ Tae‐Sung et al.^[^ ^43^ ^]^ reported a hydrochar yield of 63 wt% after 120 min at 220 °C in contrast to the 51 wt% in this study. However, Tae‐Sung et al.^[^ ^43^ ^]^ conducted their experiments with sawdust, which is more accessible than massive WBs. Mendoza Martinez et al.^[^ ^42^ ^]^ treated 4 lignocellulosic biomasses for 180 min at different temperatures, one of which is 220 °C, to quantify the effect of the reaction conditions on the products. Both, Tae‐Sung et al.^[^ ^43^ ^]^ and Mendoza Martinez et al.,^[^ ^42^ ^]^ recorded yields of well above 50 wt% after 180 min retention time at 220°C for different types of wood, which means the preparation of wood to form WBs may also have an influence on the hydrochar yield.

Further information on the composition of the surface layer of the WBs can be gathered by removing it and determining the elemental composition. As the char is very brittle, no further processing like grinding is needed. The elemental composition of the surface of the WB and D after HTC is shown in **Table** **1**.

**Table 1:** Elemental composition of the original biomass and the solid products after HTC (dry basis).

Sample	C [wt%]	H [wt%]	N [wt%]	O [wt%]
Raw	45.3 ± 0.6	6.2 ± 0.0	0.1 ± 0.0	48.4 ± 0.6
WB‐0‐W	60.4 ± 8.6	6.2 ± 0.8	0.3 ± 0.1	32.1 ± 9.4
WB‐0‐NW	56.3 ± 2.2	5.7 ± 0.1	0.3 ± 0.1	37.6 ± 2.3
WB‐30‐W	55.4 ± 3.4	5.9 ± 0.3	0.2 ± 0.0	38.5 ± 3.6
WB‐30‐NW	57.2 ± 5.2	5.9 ± 0.8	0.2 ± 0.0	36.6 ± 5.9
WB‐60‐W	60.7 ± 7.1	5.9 ± 0.8	0.2 ± 0.0	33.2 ± 7.8
WB‐60‐NW	55.1 ± 6.3	5.5 ± 0.6	0.2 ± 0.0	39.2 ± 6.9
WB‐120‐W	59.4 ± 4.1	5.5 ± 0.4	0.2 ± 0.0	35.0 ± 4.3
WB‐120‐NW	57.9 ± 4.5	5.6 ± 0.4	0.2 ± 0.0	36.4 ± 4.8
WB‐240‐W	60.8 ± 7.9	5.5 ± 0.7	0.2 ± 0.0	33.5 ± 8.6
WB‐240‐NW	59.7 ± 6.9	5.5 ± 0.7	0.2 ± 0.0	34.6 ± 7.5
WB‐480‐W	64.3 ± 0.3	5.1 ± 0.0	0.2 ± 0.0	30.4 ± 0.3
WB‐480‐NW	65.0 ± 0.1	5.2 ± 0.0	0.3 ± 0.0	29.6 ± 0.1
WB‐960‐W	63.9 ± 0.2	5.1 ± 0.0	0.2 ± 0.0	30.9 ± 0.1
WB‐960‐NW	66.0 ± 0.1	4.9 ± 0.0	0.2 ± 0.0	28.9 ± 0.1
D‐0‐W	66.3 ± 0.3	5.2 ± 0.1	0.4 ± 0.1	28.0 ± 0.3
D‐30‐W	66.5 ± 0.6	5.3 ± 0.1	0.4 ± 0.1	27.9 ± 0.6
D‐60‐W	66.7 ± 0.1	5.5 ± 0.1	0.4 ± 0.1	27.4 ± 0.2
D‐120‐W	66.4 ± 2.7	4.9 ± 0.3	0.2 ± 0.0	28.5 ± 3.0
D‐240‐W	67.7 ± 0.1	5.1 ± 0.0	0.2 ± 0.0	27.1 ± 0.0
D‐480‐W	68.6 ± 0.1	5.2 ± 0.0	0.3 ± 0.0	25.9 ± 0.1
D‐960‐W	67.5 ± 0.0	5.0 ± 0.0	0.2 ± 0.0	27.3 ± 0.0

Large deviations between the repetitions of the experiments lead to a larger estimated error, which can be assigned to the inconsistent character of the biomass as well as the differences in between the layers of the char scratched from the surface.

As expected, the C content (wt%) of all samples after the hydrothermal treatment increased compared to the raw material. As the retention time progresses, an increase of the C an N content (wt%) was found to the detriment of the H and O content (wt%). While for short retention times (0 and 30 min) the C content (wt%) were comparable, it finally remains constant at around 64 wt% after 480 min, which is close to the 66.7 wt% calculated by Jung et al.^[^ ^19^ ^]^ for total dehydration of fructose. As expected, no S (wt%) was found in the raw and converted WB and the ash content (wt%) in the WB was negligible.

To evaluate the possible influence of the different adsorbed by‐products on the surface of the converted WBs, they were washed with hot demineralized water. As expected, the C content (wt%) of both, the WB‐W and the ones without any post‐treatment, differs to a certain degree. However, contrary to what might have been expected, the WB‐W show a higher C content (wt%) than the WB‐NW. After 60 min of retention time, all the WB‐W samples exhibited a higher C (wt%) than their analogous WB‐NW samples until the end of the treatment. On the other hand, the H and O contents (wt%) decreased with increasing retention time in both, the WB‐W and WB‐NW. The N content (wt%) of the WB‐W and WB‐NW after HTC is slightly higher compared to the raw material. As already reported for the WBs without post‐treatment, the C content (wt%) of the WB‐NW ceases at 64 wt% after 480 min. Furthermore, the ash content (wt%) of the WB‐W and WB‐NWs increases with the retention time but starts to decrease slowly after 240 min.

In contrast to the WB samples, the C content (wt%) of the D samples do not achieve the “steady state” after 120 min. While, before 120 min retention time, the C content (wt%) stays approximately the same, a slight increase up from 66–67 wt% to 67–68 wt% occurs. The H content (wt%) of the D samples behave similar like in the WB samples with increasing retention time. In contrast to the before mentioned contents, the N content (wt%) might be slightly higher than both, the raw and converted WBs, even though a trend toward 0.2–0.3 wt% is observed for longer retention times.

Throughout the experiments for shorter retention times, the D samples from the ground of the reactors show approximately the same elemental contents, around 66 wt%. Nonetheless, the C content (wt%) of the D samples for longer retention times is 68 wt%, which is higher than that of the WB samples.

#### 2.3.2 Fourier Transform Infrared Spectrometry (FTIR)

**Figure 8:**
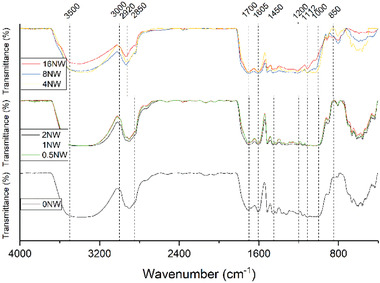
ATR‐FTIR spectra of the surface of the wooden balls (not washed) after retention times of 0 min to 960 min of HTC at 220 °C.

**Figure 9:**
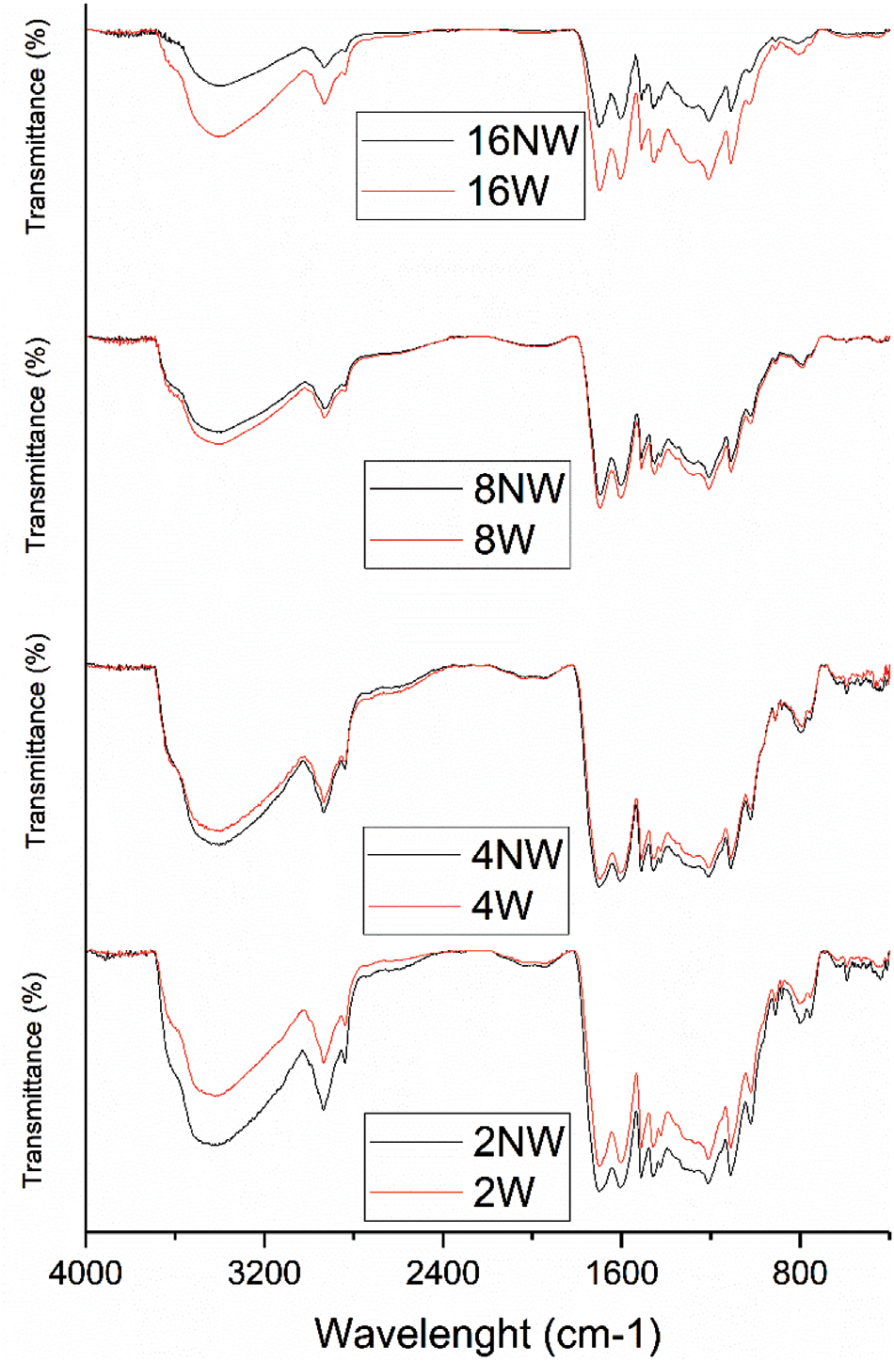
ATR‐FTIR spectra of the Ds after retention times of 0–960 min of HTC at 220 °C, each washed (W) and not washed (NW).

The Fourier transform infrared (FTIR) spectra of the WB and D after HTC are depicted in **Figure** **8** and **Figure** **9**, respectively. The effect of the different retention times can be predominantly seen in the diagram of the D samples, while it is inferior in the diagrams of the WB samples.

In Figure 8, the FTIR spectra of the converted WB‐NWs are displayed, the FTIR spectra of the converted WB‐Ws are shown in Figure S6 in the Supporting Information. The peaks of the wavenumbers between 3500 and 3000 cm^−1^, which are associated to the hydroxyl groups and carbonyl groups, are similar for all the outer covers except for the ones after 960 min of HTC. The peaks obtained at 2922 and 2850 cm^−1^ could be assigned to the ─CH stretching and aromatic ─C─H bending vibrations, however, for 960 min retention time these two peaks appear as a single peak. In Figure 8, the peaks obtained at 1705 and 1605 cm^−1^ are associated to the ─C═O of the carboxyl groups and, except for the 0 and 30 min WB‐W, the intensity of these peaks is similar for all the hydrochars. At $\tilde{\nu }$  =  1560 cm^−1^, the peak is also associated to the aromatic structure, the so‐called aromatic finger. By increasing the retention time, the intensity of the peak is reduced. The peaks at 1200 and 850 cm^−1^, whose intensity is reduced for longer retention times, can be assigned to the ─C═ O (ester) or C─O─C (ether) vibrations as a result of the decomposition of the original lignin in the raw WB. The decrease of the peak intensity from 800 to 400 cm^−1^ is referred to in previous studies^[^ ^45^ ^]^ as a consequence of the degradation of the aromatic rings of the lignin contained in the initial WB.

To find out more about the nature of the Ds, Figure 9 shows the related FTIR spectra.

In Figure 9, the FTIR spectra of the Ds after HTC is displayed. By increasing the retention time, the peaks at wavenumbers between 3600 and 3200 cm^−1^ decrease, which proves the loss of –OH groups. In comparison to the WBs, the Ds have only one peak at 2950 cm^−1^, which is the same as for the WBs after 960 min. The signals between 1950 and 1200 cm^−1^ prove that the Ds are mainly made of the same functional groups as the WBs. Between 1705 and 1605 cm^−1^, the aromatic C–O signals are less intense than of the WBs, which is also true for the signals around 1560 cm^−1^ and those between 1200 and 800 cm^−1^ (aromatic ester, ether and hydroxyl groups). Still, for short retention times, the intensity is close to that of the WBs but for longer retention times all signals are less intense.

Compared to the spectra of the charred surfaces of the WBs, there are only minor differences to the Ds.

#### 2.3.3 Quantification of the Liquid Phase By‐products

**Figure 10:**
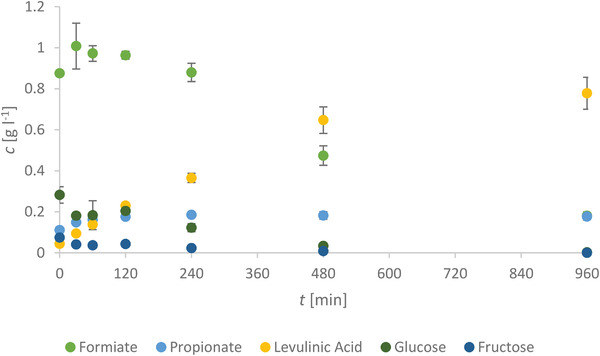
Evolution of the concentration of different acids and sugars over the course of the reaction.

**Figure 11:**
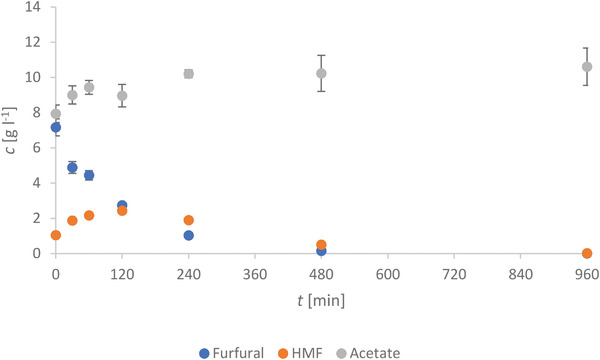
Evolution of the concentration of Furfural, HMF and Acetate over the course of the reaction.

Finally, the liquid products of the HTC were analyzed by HPLC to find out about the compounds in the liquid phase. In **Figures** **10** and **11**, the concentrations of the chemical compounds present in the process water after the HTC treatment (Tables S3 and S4, Supporting Information) are displayed versus the retention time. All displayed results are the mean concentrations of two samples held at 220 °C for the same time.

The chemical compounds formed with increasing time at 220 °C by the degradation of wooden material were previously studied.^[^ ^46^, ^47^, ^48^, ^49^ ^]^ In general, a low starting concentration of sugars, which decreases for longer retention times, is expected for a wooden material. The maximum of the HMF concentration is reached around 120 min, while the furfural concentration is higher at shorter retention times with the highest concentration at 0 min. First, the formation of furfurals from wood is fast and it is shown by the production of around 7.5 g L^−1^ by just heating up to the desired temperature. Second, the formation of HMF might be slower but this offers a broader range of retention times to tailor the products, e.g., longer retention times if acids are the desired product.

#### 2.3.4 GC–MS

**Figure 12:**
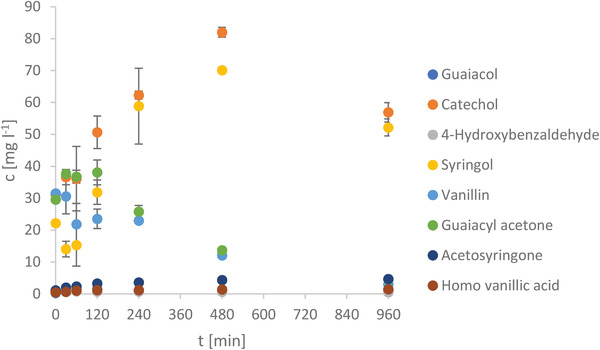
Concentration of chosen compounds of Lignin degradation with increasing retention time determined by GC‐MS.

In order to find out about the conversion and effect of lignin, the concentration of the chosen compounds with increasing retention time is displayed in **Figure** **12**. After determining the compounds with the highest concentrations among those from previous studies,^[^ ^50^ ^]^ the following 8 compounds were chosen: 2‐methoxyphenol (Guaiacol), catechol, 4‐hydroxybenzaldehyde, 2,6‐dimethoxyphenol (Syringol), 4‐hydroxy‐3‐methoxy benzaldehyde (Vanillin), 4‐hydroxy‐3‐methoxy phenylacetone (Guaiacyl acetone), 4′‐hydroxy‐3′,5′‐dimethoxy acetophenone (Acetosyringone) and homovanilllic acid.

Among the compounds with the highest concentrations in previous experiments, the highest concentrations in the current experiments are provided by Catechol, Syringol, Vanillin, and Guajacylacetone. Most of the compounds’ starting concentration is close to the concentration after 960 min, while in between the concentrations mostly rise before they decrease again. Vanillin and Guaiacylacetone evolve in a different way as they either do not rise at all or only to a small extent before the concentration starts to decrease. The highest concentrations are found at 480 min, where Catechol is at around 80 mg L^−1^ and Syringol at ≈70 mg L^−1^.

## 3 Discussion

### 3.1 Outer Appearance of the WBs

All HTC experiments show the brown color of biomass turning black with increasing retention time at a certain temperature and the reason stated is the degradation of the biomass to form new compounds, both liquid and solid.

The solid product, referred to as char, can be linked to two processes: the direct solid‐to‐solid conversion forming primary char and/or the (poly‐)condensation and re‐polymerization of the degradation products, forming so‐called secondary char.^[^ ^5^ ^]^

**Figure 13:**
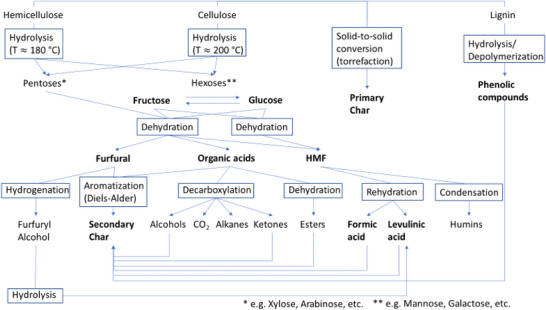
Reaction scheme of the hydrothermal carbonization of lignocellulosic biomass, actually proofed products are highlighted in bold letters; compiled according to.^[^ ^51^, ^52^, ^53^, ^54^, ^55^ ^]^

A short consideration of the available processes for solid‐to‐solid conversion reveals the absence of oxygen to be an important factor. Together with the parameters of the hydrothermal treatment reported, only torrefaction would be left to be considered. While torrefaction needs no water, the hydrothermal degradation of biomass by hydrolysis needs an excess of water. The changes in the outer appearance of the WBs thus can be associated mainly to the chemical reactions, which take place during the hydrothermal treatment according to **Figure** **13**.^[^ ^51^, ^52^, ^53^, ^54^, ^55^ ^]^

First, the degradation of the biomass starts with the hydrolysis of the glycosidic bonds of the carbohydrates. As the glycosidic bonds of hemicellulose are less stable due to their branched, noncrystalline structure, the hemicellulose starts to degrade at lower temperatures than cellulose to form mainly pentoses.^[^ ^47^, ^56^, ^57^ ^]^ The following step is the degradation of cellulose at higher temperatures by hydrolysis to glucose (Figure 10). At the same time, the dehydration of the sugars (xylose, glucose, arabinose, mannose, etc.) originating from hemicellulose occurs, e.g., to form acetic acid (Figure 11). While the degradation of the biopolymers is a kinetically slow step (rate determining step), the conversion of sugars to consecutive products is faster according to Paksung et al.^[^ ^25^ ^]^ Therefore, the hexose (glucose) and its tautomerism product fructose are also further processed by dehydration (Figure 10). Via dehydration, up to three water molecules can be split‐off from the sugar molecules to form furfurals like HMF (Figure 11).^[^ ^58^ ^]^ The furfurals can perform aromatization, e.g., by Diels‐Alder reactions in the presence of a dienophile, which also could be furfurals. On the other hand, acids are formed by the dehydration and partial rehydration of hexoses (Figures 10 and 11).^[^ ^59^ ^]^ This is a two‐step (acid‐catalyzed) reaction, which includes the rehydration of the previously formed HMF to levulinic acid (Figure 10).^[^ ^60^ ^]^ Moreover, these acids include industrially important species like acetic, formic and lactic acid (Figures 10 and 11).^[^ ^61^, ^62^, ^63^, ^64^ ^]^ These acids are one of the main reasons why all the reactions are self‐accelerated as the formation of acids increases the availability of protons, which are needed or advantageous for reactions like dehydration, (poly)condensation and aldol reactions.^[^ ^65^, ^66^ ^]^ At the same time, the pH influences the formation and degradation especially of HMF.^[^ ^59^ ^]^ Furthermore, the acids are converted by decarboxylation to form intermediates like simple Alkanes together with CO_2_, under the premise of an –I or –M group in C*_α_* position to the carboxylic group, or alcohols. In addition, the dehydration to form esters, CO and H_2_O occurs.^[^ ^55^, ^67^, ^68^, ^69^ ^]^ As already mentioned, the acids cause the degradation of HMF, which leads to aromatic compounds via aromatization or also results in the formation of hydrochars as well as levulinic acid (Figure 10).^[^ ^6^, ^70^ ^]^

The chemical compounds formed with increasing time at 220 °C by the degradation of woody material were previously studied.^[^ ^46^, ^47^, ^48^, ^49^ ^]^ A low starting concentration of sugars, which decreases for longer retention times, is expected for a wooden material. This is largely due to the structure of the wood, which hinders the degradation of cellulose and hemicellulose into simpler carbohydrates (i.e., glucose, fructose, mannose, xyloses) by the rupture of glycosidic bonds by hydrolysis (Figure 13). Indeed, considering the impact of the degradation of (by‐)products (HMF, furfural) obtained in this process from a biorefinery point of view, the concentrations of HMF and furfurals become more interesting (Figure 11). The maximum concentration of HMF is reached at around 120 min, while the furfural concentration is higher at shorter retention times with the highest concentration at 0 min. This is in accordance with the previous study of Körner et al.,^[^ ^59^ ^]^ who suggested that such results might be a consequence of two different pathways. First, the formation of furfurals from wood is fast due to the production of around 7.5 g L^−1^ of liquid by just heating up to the desired temperature. The fast decline of the furfural concentration is also predictable by taking the high amount of acids into consideration. Those acids force the hydrogenation of the furfurals to furfuryl alcohol. As shown before,^[^ ^5^ ^]^ acids catalyze the degradation of biomass and products, therefore, receiving such high concentrations of HMF means a high turnover rate from sugars to HMF. This is also supported by the low concentrations of sugars, which is expected for the chosen reaction temperature (Figure 10).

A special case is the conversion of lignin, which is harder to accomplish as the structure of lignin is polyphenolic and not uniform like cellulose. Lignin consists of monomers connected by polar C─O and nonpolar C─C bonds.^[^ ^71^ ^]^ This is important concerning the reaction as mainly the polar oxygen containing bridging bonds can be affected by water.^[^ ^71^ ^]^ The split of the C─C─ bonds can only occur by heat, like, e.g., in a dry conversion.^[^ ^71^ ^]^ Nonetheless, lignin is slowly converted to phenolic compounds, which to a minor extent is also possible for the parameters applied in this study.^[^ ^72^ ^]^ Those phenolic compounds are assumed to also become incorporated into the hydrochar.^[^ ^73^ ^]^ In this case, the char would partially consist of (poly‐) phenolic compounds like Guaiacylacetone and Vanillin found at medium retention times at high amounts as shown in Figure 12. Interesting to note is all the concentrations decrease after a certain retention time, which means they vanish from the liquid. However, depending on the compound, their incorporation into the char seems to work at different rates. Some are consumed at a high rate (e.g., Homo vanillic acid, syringol), so their concentration stays at a very low level. Others’ concentrations only decrease from a mediocre starting concentration and again others’ concentrations increase at short retention times to decrease for longer retention times. For all pathways it needs to be considered that the WBs are industrially produced, thus pretreated, which could lead to a slightly different behavior than a totally natural product like, e.g., driftwood.

In Figure 4, wherein the thin layer on the surface of the WBs can be clearly seen, the darkening color of the surface of the WBs points to reactions that start after some time, as its precursors (acids, furfurals, etc.) need to be produced first. As described in Figure 13, the products of the HTC can (poly) condensate to form a solid residue, the secondary char. This is supported by the SEM picture of the char surface in Figure 6. Here, the typical spheres, which are a result of the polycondensation during HTC, are shown on top of the original or charred biomass, especially the pyrochar. This is a result of heating the WBs up to the reaction temperature as degradation of the biomass already starts below 200 °C.^[^ ^74^ ^]^ With increasing retention time, the amount of hydrochar increases by forming a larger number of spheres (Figures S1–S4, Supporting Information). This is supported by the images of the longer retention times, where the density of spheres increases additional to spheres growing on a wavelike surface (Figures S1e–S4, Supporting Information). While at 120 min, no clear distinguishable spheres could be found, the growth process of the hydrochar gets accessible especially at 480 min because of the oval form of the particles with a structured surface (Figure 7). A reason for this deformation could be the high number of precursors in the liquid phase which immediately attach to possible sites of the surface. Another possibility for this kind of behavior is Ostwald ripening where small particles get consumed by bigger particles, probably in return for their clear shape.^[^ ^75^, ^76^ ^]^ The longest retention time reveals cratering of the surface, remembering of the porous forms of rust.^[^ ^77^ ^]^ The complete surface of these samples is covered by more or less distinguishable spheres on top of a layer of molten like spheres, resulting in a partially wavelike, partially porous surface. Along the former wooden fibers, the layer thickness and outer appearance of the char is displayed.

Large deviations between the repetitions lead to a larger estimated error, which can be assigned to the inconsistent character of the biomass as well as the differences in between the layers of the char scratched from the surface. Therefore, the composition of the samples sometimes varies to a larger degree (Table 1). As expected, the C content (wt%) of all samples after HTC increased, as water got split off by dehydration. This is further supported by a decreasing H and O content together with an increasing C content (wt%) with increasing retention time. However, the dehydration reactions are not terminated as glycosidic bonds still remain even after 960 min.

Finally, the C content (wt%) remains constant at around 64 wt% after 480 min, which is close to the 66.7 wt% calculated by Jung et al.^[^ ^19^ ^]^ for total dehydration of fructose. Furthermore, this means that no or only few decarboxylations took place. Jung et al.^[^ ^19^ ^]^ also proposed that the carbon content would increase to above 70 wt%, which could not be observed here.

In comparison to the raw WB, the WBs after HTC exhibit higher C and N content (wt%), but lower H and O content (wt%). In those WBs produced at 0 min and 30 min retention time, the composition of the S‐WB is similar, so it was assumed that the degradation by hydrolysis and dehydration is still slow in the beginning, thus leading to a small amount of precipitate on the ground of the autoclave. This means that, at least in the beginning, torrefaction is the dominant conversion. After 120 min, the carbonization reaction was completed, highlighted by the dissipation of the transition layer (Figure 4), and independent of any further retention time between 120 and 960 min. Thus, the C content (wt%) stays similar. It is assumed, as the C content (wt%) did not increase abruptly, that the carbonization of the outer shell was already completed after 60 min retention time. This means that, according to the assumptions made, enough compounds from the liquid phase precipitated on the surface of the WBs to cover the complete surface. Afterward, the composition will not change anymore or only slightly as the same compounds participate again and again. In the end, a layer of hydrochar will remain to cover the WB while every further precipitate sinks to the ground as highlighted by an increasing thickness of the D. As already shown in previous studies,^[^ ^78^, ^79^, ^80^, ^81^ ^]^ consecutive reactions like the aldol condensation and β‐elimination lead to a decrease of the hydrogen content as water is released.

By considering the FTIR spectra, two things are interesting. First, the relative intensity of hydrophilic functional groups like hydroxyl groups and hydrophobic functional groups like double bonds stays relatively equal for all hydrochars on the surface throughout all the different retention times. Thus, no change in the ratio of these functional groups takes place after the degradation begins, the maximum hydrophobicity is achieved after short retention times. Second, the hydrophobic character of the Ds increases with increasing retention time as the hydroxyl signal gets less intense. This could be due to the ongoing carbonization resulting from the contact with the reactor wall.

To get rid of possibly adsorbed compounds on the surface of the converted WBs, three out of six were washed with hot demineralized water. As expected, the C content (wt%) of both, the WB‐W and the ones without any post‐treatment, differ to a certain degree. However, different from what might have been expected, the WB‐W exhibit a higher C content (wt%) for short retention times than the WB‐NW. Thus, the washing does not consistently lead to a decrease but an increase as the new formed outer layer would have to still undergo further condensation to increase the C content (wt%) by water elimination to the same level exhibited by the rest of the converted WB (e.g., transition layer, pyrochar). With increasing retention time, the trend is inverted as from 480 min the WB‐NW exhibits the higher C content (wt%), which could point to a possible formation of oligomers in the liquid, which finally precipitate. Nonetheless, this is a clear indication that there are some compounds left on the surface of the converted balls, which can be washed out. The draining of components which were released from the WBs is quite fast, otherwise they would have been detected to a higher extent.

This supports the idea that the compounds attached to the surface, which built the new outer layer, are only loosely bound. Furthermore, the color of the liquid after separation is free from particles and thus turned from opaque to clear, indicating the formation of polymeric compounds which did not precipitate yet.

Basically, the same behavior as for the C content (wt%) is found in the case of the O content (wt%) considering the washing step of the WB‐W and WB‐NW. In the beginning, the O content (wt%) of the WB‐W is well below the WB‐NW, which is inverted from 480 min on. One exception, the WBs after 30 min, show a higher O content (wt%) for the WB‐W before. As the O content (wt%) is calculated according to Equation (2.1), the differences mainly are related to the differences in the C content (wt%) as the other elemental contents differed only slightly and the calculated errors of the C content (wt%) are large compared to those of the H and N content (wt%). However, a generally decreasing O content (wt%) is a result of the dehydration and decarboxylation taking place, also supported by a decreasing H content (wt%). These reactions are not faster on the outer layer due to the contact with water. In contrast, the reactions below the outer layer are faster. The reason might be the high availability of acids as discussed before (Figure 10). By assuming precipitation of previously solved compounds (i.e., HMF) on top of the WBs during the reaction, the degree of degradation by decarboxylation and dehydration of the layer below this loosely attached precipitate could be explained. On the other hand, the H content (wt%) decreased with increasing retention time in both, the WB‐W and WB‐NW due to the same reactions discussed for the O Content (wt%). Furthermore, the N content (wt%) of the WB‐W and WB‐NW after HTC is slightly higher compared to the raw material. The studies carried out by Xiao^[^ ^82^ ^]^ and Kang^[^ ^83^ ^]^ show an opposite effect related to the N content (wt%).

Additionally, the sense of Ostwald ripening gets fortified by considering Figure 7, which shows the comparison of a WB‐W sample and a WB‐NW sample after the separation of solid and liquid. While it may be hidden by loose particles and pieces of char in the converted WB‐NW for 480 min, the spheres are clearly distinguishable in the WB‐W for 480 min due to the reduction of loose particles by washing. The formation of loose particles could be caused by a plethora of reasons. Some of them are weak intermolecular forces, so an adhesion or even binding is not favorable, loosening of smaller particles by Ostwald ripening or too slow covering of pyrochar by hydrochar leading to a thin layer on the surface of the pyrochar instead of a closed shell around it.^[^ ^24^, ^75^, ^76^, ^84^, ^85^ ^]^ Moreover, this is further indication on the maximum thickness of the outer layer as well as the on‐going condensation of, e.g., HMF. More “spheres” together with the surface structure in the comparison of WB‐W and WB‐NW in Figure 7 point out that part of the surface layer can be removed while the total number of spheres still increases with increasing retention time.

However, considering the changes underneath the surface of the WBs, layer formation has to be discussed as proposed in Figure 1. With increasing retention time, Figure 4 showed a thin layer on the surface of the WB and a growing layer from the core of the WBs, together with a vanishing transition layer in between. This is a clear indication that the outer layer mainly consists of hydrochar, formed by the polycondensation of degradation products from the liquid, as it is the only part of the WBs which is in direct contact with the liquid phase, and is limited in its thickness. As the outer layer also becomes brittle with increasing retention time, the following mechanisms could be proposed – an on‐going condensation of the layer, which could be proofed by an increasing C content (wt%), together with the precipitation of the newly formed products as soon as the maximum thickness of the outer layer is reached.

Underneath the outer layer, the transition layer changes with increasing retention time (Figure 4). As the transition layer vanishes before reaching 120 min, only shorter retention times will be evaluated. In fact, the transition layer is clearly visible at 0 min, as a visually nearly unconverted area between the core and the outer layer. However, considering the progress over the next 60 min, the transition layer gets consumed from within the ball, therefore it could be guessed to be turning into pyrochar. The absence of water is a further indicator of torrefaction turning the WB into pyrochar from within. Further insight on the transition layer should be gathered in future experiments especially for short retention times with the focus on the interfaces. This is important considering the complete conversion and therefore the nullification of the influence of the original biomass on char properties.

In the case of an even partly charred WB, the strong interaction between the OH– groups of carbohydrates is destroyed, thus the number of these functional groups is reduced to a large degree. However, without those interactions, the material becomes brittle. This could be seen before, as the cracks in the balls turned larger and became numerous compared to shorter retention times (see Figure S5 in the Supporting Information). Together with the layer production discussed before, it seems clear that the degradation of the wood could still be extended, even after 960 min.

An unexpected part of the conversion of wood was the appearance of the precipitate on the bottom of the reactor. To evaluate this phenomenon, the Ds were analyzed according to the procedures for the WBs.

It is assumed that, even for shorter retention times, some precipitate appeared which then either was too few to form a D or had not been precipitated from the WB yet but was still loosely attached to the surface of the WB by intermolecular forces. Moreover, partial and time‐limited adhesion to the surface of the WB could prevent the newly formed precipitate to sink to the bottom of the reactor.

As the precipitate gets in contact with the hot reactor bottom, it might stay in a molten, high viscous but liquid state, which fits and sticks to the reactor after cooling. This D was then taken out of the reactor by loosening it from the bottom with a spatula, which is also the reason why all the Ds broke because of the very brittle condition they were in. The top of the D, which is covered by the liquid phase, shows a glossy, plastic color, which can be due the precipitation of the polymerized intermediates. This precipitation takes place by the sinking of the cover plus elimination of water during (poly‐)condensation, thus forming a polycondensate. The bottom of the D shows a color similar to charcoal, which might be due to the walls of the reactor being hotter than the reaction medium because of the losses of heat due to convection during the HTC process and conduction during the quenching of the reaction. Furthermore, the WBs were partially in contact with the gas phase in the reactor, which also exhibits different heat transport than water, before they sunk to the ground. Another possibility is the change in properties of water by increasing the temperature and pressure during the reaction which led to the sinking of the WBs.

Moreover, the thickness of the D increases with increasing retention time. This may be due to four reasons: 1) The secondary char forms on the surface of the WB as new outer shell, loosely attached via intermolecular forces before it precipitates and becomes a D. 2) The polymers formed in the liquid phase precipitate directly due to the acidic pH of the aqueous medium and get carbonized because of the temperature. Thus, the Ds would be carbonized humins. 3) The quenching of the reactor can precipitate polymers formed in the gas‐liquid interface of the reaction medium. 4) Torrefaction inside of the WB occurred and formed oily compounds, which gathered on the surface of the WB. These oily compounds were then washed off and agglomerated on the particles formed by 1).

The Ds from the bottom of the reactors throughout the experiments show for shorter retention times approximately the same elemental contents, around 66 wt%. This means, that it is always the same precipitation taking place. Nonetheless, the C content (wt%) of the Ds for longer retention times is 68 wt%, which is higher than that of the WBs. Here, the post‐treatment also shows little influence, which is obvious as the Ds exhibit a large surface which is in touch with the water. Furthermore, for precipitation, the compounds must sink from the balls which float at the surface of the water at the beginning. This means that the density of the disc is larger than that of water or that an agglomeration took place on crystals on the walls. However, as the D only appeared on the floor of the reactor, it seems unlikely that crystals only grew there but not on the rest of the reactor walls. As there is no difference throughout the different reaction conditions, the sinking of the balls seems to have no influence on the precipitate.

The C content (wt%) does not achieve the “steady state” after 120 min as for the WB, which might be a consequence of direct contact with the metal wall of the reactor (if precipitation is assumed since the beginning) or that the initial carbon particles released in the aqueous phase during the hydrolysis of the WB are the seeds on which the aromatic compounds precipitate. While before 120 min retention time, the C content (wt%) of the Ds stays approximately the same, a slight increase up from 66–67 wt% to 67–68 wt% occurs. This might be due to the ongoing polycondensation reaction, especially of the areas in contact with the walls, which eliminates water. This, in turn, is supported by the H content (wt%) of the Ds, (Table 1) which behaves similar to those in the WBs with increasing retention time and might be due to on‐going dehydration or polycondensation. In contrast to the before mentioned contents, the N content (wt%) might be slightly higher than both, the raw and converted WB, even though a trend toward 0.2–0.3 wt% is observed for longer retention times. Thus, the N content (wt%) is only affected by the HTC in this case, which in turn means that both, the outer cover of the WBs and the Ds have a comparable N content (wt%) and supports the idea that both are the same material.

In Figure 8, the FTIR spectra of the converted WBs are displayed. The peaks of the wavenumbers between 3500 and 3000 cm^−1^, which are associated to the hydroxyl groups and carbonyl groups, are similar for all the outer covers except for the ones after 960 min of HTC. This suggests that a similar degree of dehydration took place during the degradation of the WBs. Therefore, the lower intensity of these peaks for 960 min retention time can be associated to the loss of carboxyl groups with longer retention times. The peaks obtained at 2922 and 2850 cm^−1^ could be assigned to the ─CH stretching and aromatic ─C─H bending vibrations and confirm that aromatization took place during HTC of the WB, however, for 960 min retention time these two peaks appear as a single peak. This may be a hint for total aromatization of the solid if the retention time is chosen long enough. In Figure 8, the peaks obtained at 1705 and 1605 cm^−1^ are associated to the ─C═O of the carboxyl groups and, except for the 0 and 30 min WB‐W, the intensity of these peaks is similar for all the hydrochars. Thus, the decarboxylation on the surface of the WB should be finished. At $\tilde{\nu }$  =  1560 cm^−1^, the peak is also associated to the aromatic structure, the so‐called aromatic finger. By increasing the retention time, the intensity of the peak is reduced. The reduced intensity of the peaks at 1200 and 850 cm^−1^ for longer retention times, can be assigned to the ─C═O (ester) or C─O─C (ether) vibrations as a result of the decomposition of the original lignin in the raw WB. The decrease of the peak intensity from 800 to 400 cm^−1^ is referred to in previous studies^[^ ^45^ ^]^ as a consequence of the degradation of the aromatic rings of the lignin contained in the initial WB.

Along with a broad signal between 2500 and 3600 cm^−1^ representing the carboxylic groups with strong hydrogen bonds to hydroxylic groups, there are a lot of indicators for aromatic groups presented. This is in good accordance with the assumption that the surface of the WBs is converted to char, either hydrochar due to the surface groups or pyrochar with adsorbed acids. However, a combination of both chars is the likeliest solution. Peak intensities of the surfaces of the different chars differ to a large extent especially between the shortest and longest retention times. Since the decrease of the signals is directly related to the retention times, the highest influence on the surface of the char can be assigned to the hydrothermal reaction mechanisms, while the washing of the samples during the char collection, has a comparatively minor influence. Nonetheless, the differences between washed samples and those, which were not washed, is still considerable. Albeit the spectra of the washed samples express a lower intensity for the reactions of 120 and 240 min, this characteristic is opposite for the reactions for 480 and 960 min, where the washed samples display a lower intensity compared to the ones which were not washed. This behavior may be explained by the char forming mechanisms. While the pyrochar formation occurs from within the biomass, directly converting solid biomass to solid char, there are strong interactions between both parts. A dissolution of pyrochar from the biomass seems to be hard to accomplish. In contrast, hydrochar is built from solved intermediates in the liquid, precipitating on the surface of the biomass, thus the hydrochar should be easier dissected and released by the surface.^[^ ^48^ ^]^ Another possibility would be that the separated layer mainly consists of humins or aromatic compounds.

To find out more about this, Figure 9 shows the FTIR spectra of the Ds on the bottom of the reactors, which probably are made of the same material. The effect of the different retention times is predominantly seen in the diagram of the Ds and only to a minor degree in the diagram of the balls. By increasing the retention time, the peaks at wavenumbers between 3600 and 3200 cm^−1^ decrease, which proofs the loss of –OH groups. In comparison to the WBs, the Ds have only one peak at 2950 cm^−1^, which is the same as for the WBs after 960 min. This can be due to the previously mentioned hypothesis that the D is formed from aromatic compounds. The signals between 1950 and 1200 cm^−1^ proof that the Ds are mainly made of the same functional groups as the WBs. Between 1705 and 1605 cm^−1^, the aromatic C–O signals are less intense than of the WBs, which is also true for the signals around 1560 cm^−1^ (aromatic finger) and those between 1200 and 800 cm^−1^ (aromatic ester, ether and hydroxyl groups). Depending on the reaction mechanism and as some of the functional groups could still be degraded by water, the intensity of those signals is lower. For short retention times, the intensity is close to that of the WBs but for longer retention times all signals are less intense, which supports the idea of degradation over time. A higher aromaticity coupled with less functional groups explains the brittle character of the Ds and outer shell of the converted WB, as the molecular interaction decreases due to a loss of hydrogen bridges and dipolar interactions.

Compared to the spectra of the charred surfaces of the WBs, there is no difference to the Ds. Together with the points mentioned before and the increasing thickness of the D layer with increasing retention time, it might be concluded that the Ds consist of the same material as the surface of the WBs. Possible reasons are repelling intermolecular forces on the surface of the WB which would lead to the conclusion that only a certain number of products can form and (poly‐)condensate on the surface of the WBs. Furthermore, liquid products like 5‐HMF, which are transported away from the WBs due to concentration gradients could (poly‐)condensate due to the higher temperatures close to the walls, which also supports crosslinking to form a polycondensate. This would also explain the brittle character of the Ds.

### 3.2 Inner Layers

According to the hypothesis from previous studies,^[^ ^9^, ^10^, ^19^, ^86^, ^87^ ^]^ the outer layers of the WBs are formed by the adsorbed intermediate compounds (i.e., furans) that are present in the aqueous phase due to the hydrolysis, dehydration, and aldol‐reactions. Thus, the soaked water is trapped inside the WBs, where torrefaction should be the more important mechanism compared to HTC. This idea agrees with the previous study of Paksung et al.,^[^ ^25^ ^]^ which showed that during the HTC of cellulose both, hydrolysis and pyrolysis, take place. However, the inner core also is black, which means that the HTC of the outer shell is actually not the only process that takes place during conversion. One aspect to consider is the soaked water, which leads to a different heat transfer in the inner of the WB, at least close to the surface, compared to a dry one where air is the worse heat transfer medium.^[^ ^9^ ^]^ Thus, one possibility includes that the whole WB should always have nearly the same temperature because the good heat transfer by water distributes the heat homogeneously in the ball. Another factor is the degradation by the influence of the water which probably penetrated inside of the WBs. This means, in the inner core of the WB hydrolysis and probably dehydration convert the former wood into sugars, HMF, acids and water, thus creating a gradient radially pointing to the outside of the WB. Covered by an already formed char layer limiting the exchange with the surrounding medium, the degradation of the layers between the inner core and outer shell of the particle could be suppressed due to a huge amount of the product molecules. As the brighter area below the surface disappears after 120 min, the conversion of this area goes on. As a consequence, the whole profile turns darker with increasing retention time. However, no proof could be found as to how deep the water penetrates the WB, even though cutting soaked balls by half.

Another possibility to compare the effect of the conversion inside of the WBs is pyrolysis, especially considering the fact that char produced by solid‐to‐solid conversion is often referred to as pyrochar. Pyrolysis is a high‐temperature process to convert biomass into gas, oils, other liquids and solids while under inert atmosphere.^[^ ^88^ ^]^ Despite the present process conditions, neither the temperature is high enough (>300 °C) nor the atmosphere is inert (traces of oxygen even in water). This is the reason to refer to torrefaction instead of pyrolysis, as the temperature is lower for torrefaction (<300 °C) while the process is comparable.

A formation of char without the influence of water in the core by torrefaction would lead to a rather uniform formation of pyrochar. As discussed for the transition layer, it would be interesting to further evaluate the material in the core, especially the interface between the core and the transition layer to find out what happens here. If actually only pyrochar is produced, the material in the core could be an interesting starting point for tailor‐made materials. Moreover, if most of the hydrochar either precipitates or could be washed off, two separate materials could be produced and separated by one single process.

### 3.3 Implementation in Future Processes

In the view of the separation of products from the process water and from the solids, the ratio between hydrochar and pyrochar is of great importance. While pyrochar adsorbs possible phosphates during the degradation and carbonization of the biomass, hydrochar does not. In an effort to separate the highest possible amount of phosphates, the equilibrium should tend strongly to hydrochars. In the context of this research, the conclusion to use lower temperatures, shorter reaction times and small/flat pieces of biomass could be drawn. However, depending on the desired products, a compromise between the amount of hydrochar and pyrochar might be necessary and to find the suitable parameters for larger pieces of biomass, this research revealed the first combinations. In addition, the correlated liquid products and the assumed network of reactions, supported by the GC/MS, further fortified and revealed new pathways and correlations of liquid and solid products. Moreover, the long term process management showed that it might be rewarding to keep the process running throughout several hours, reducing the energy costs while still producing valuable platform molecules.

## 4 Conclusion

In this study, the set temperature of 220°C ensures that the hemicellulose and cellulose are partially converted, however, the lignin remains mainly intact and leads to a deformed shape of the original balls. Structural changes in the wooden balls turned their outer appearance brittle while, underneath the surface, the structure is in between brittle for long retention times and hard for short times with some filament kind of structure in between. Especially for retention times from 480 to 960 min, the complete structure tends to fall apart by applying force. Here, the filaments were still intact, even though mainly converted, but were not sticking to each other. This would draw the conclusion that the lignin should be partially hydrolyzed, while the hemicellulose and amorphous part of cellulose would be mostly hydrolyzed. Nonetheless, it is more likely that, by growth and shrinking, the intermolecular forces decreased and with longer distances, the interaction is decreased.

Two different pathways were determined during the HTC process for the conversion of a complex biomass to hydrochar. A gradient color change from the core of the balls to their surface can be seen for short retention times. This is due to the dominant solid‐to‐solid conversion pathway in the core, which is similar to the torrefaction process. In contrast to the core, secondary char is created on the surface as it is in contact with the aqueous environment. However, in contrast to the assumption made, the hydrochar layers seem to have limited thickness. Nonetheless, the transition layer vanished continuously already after short retention times as expected.

At medium retention times, high concentrations of Guaiacyl acetone and Vanillin are found, which decrease for longer retention times meaning they are incorporated into the char, however at very different rates. Moreover, a new effect of the HTC not reported previously is shown. A disk was formed which might be assigned to the polycondesation reaction and/or to partially hydrolyzed lignin.

## 5 Experimental Section

Wooden balls (beech wood) with an average diameter (measured with a caliper) of (12.00 ± 0.01) mm, which were provided by Holz–Allerlei, were used as initial biomass. Their initial water content was determined according to the oven‐drying method for determining the water content^[^ ^89^ ^]^ by weight before and after 9 days at 105 °C in the oven and additional 2 h at 150 °C to evaporate capillary water. The sample was cooled to room temperature before weighing. After the measurement, the sample was again placed in the oven overnight. This process was repeated until the weight was constant.

### Experimental Procedure

In stainless steel (V2A) autoclave reactors with a total internal volume of 250 cm^3^ and an experimental setup previously described by Arauzo et al.,^[^ ^3^ ^]^ HTC experiments were performed. Reactors were filled with 6 wooden balls of a total weight of (18.02 ± 0.02) g and (100 ± 0.02) g distilled water. Wetting and impregnation of the balls with water was guaranteed by immersion in the water.

One reaction temperature (220°C) and six retention times (0, 30, 60, 120, 240, 480, and 960 min) at this temperature were selected for the HTC experiments. Retention time is defined as the time since the reactor reaches the desired reaction temperature. The preheating time to achieve the desired reaction temperature in the reactor, filled with biomass and water was 60 min. As the preheating time should be determined for each set of experiments, the heating of the reactor was monitored for the first set of experiments and set to 60 min due to the nearly constant time needed to reach the reaction temperature. In addition, the same set of reactor, oven, thermocouple and temperature display were used to improve the comparability and reproducibility of the experiments. After the desired retention time, the reactor was quenched in cold water for 30 min. Then, the balls and the liquid, with dissolved and precipitated material, were separated with quantitative filter grade 413 VWR filter paper (VWR European Cat, France) in a Büchner funnel. The liquid fraction was frozen at −24 °C for further analysis, while the precipitated solid residue and 3 balls of each experiment were washed using three times ≈50 mL of hot distilled water (temperature ≈70 °C). This was done to find out, if the outer layer on the surface of the converted ball is removable, as the hydrochar is formed from solved compounds out of the liquid. If the outer layer would be pyrochar formed by solid‐to‐solid conversion, there would be no possibility to remove this layer by washing. The wooden balls (WB) and the precipitate observed after HTC treatment (D) were dried at 105 °C until weight consistency was reached.

### Preparation of Solid Samples

The surface of the wooden balls (W and NW) after the HTC process was scraped with a sharp blade to obtain a homogenous dust for further solid analysis (i.e., EA, SEM and FTIR). One of the converted balls was also cut by half to get an image of the structure of the layers of the ball. The precipitated solid was ground in a CryoMill from Retsch GmbH (Haan, Germany) for 2 min at a frequency of 30 s^−1^ and stored in glass bottles.

### Analysis

For visual analysis, photos of the balls on a holder were taken with a Nikon D3400 coupled with a lens (Nikon AF‐S DX Nikkor 35 mm 1:1,8G). Position of the camera and the holder were set to guarantee the comparability of the balls.

For solid analysis, the determination of the yield of hydrochar of the balls was done according to the Equation (1)

(1) $$Hy\;\left(\mathrm{wt}\%\right)=\frac{\mathrm{mass}\;\mathrm{of}\;\mathrm{dried}\;\mathrm{hydrochar}\;\mathrm{balls}\left(g\right)}{\mathrm{mass}\;\mathrm{of}\;\mathrm{dried}\;\mathrm{balls}\left(g\right)}\;*100\%$$

The elemental composition (CHNS) of the balls and precipitates after HTC was determined by an EuroEA 3000 Serie (*EuroVector S.P.A)* equipped with a thermal conductivity detector (TCD). While the elemental compositions of C, H, N, and S are determined directly, the content of O was calculated as 100% minus the other contents including ash as highlighted by Equation (2)

(2) $$\begin{array}{c} O\;(\mathrm{wt}\%)=100\%-N\;(\mathrm{wt}\%)-C\;(\mathrm{wt}\%)-H\;(\mathrm{wt}\%)-S\;(\mathrm{wt}\%)-\mathrm{Ash}\;(\mathrm{wt}\%) \end{array}$$

The ash content was determined with a STA449 F5 Thermogravimetric analysis unit (TGA) of Netzsch. In an atmosphere consisting of 20 mL min^−1^ N_2_ as protective gas and 50 mL min^−1^ synthetic air, the samples were heated up to 900 °C with a constant heating rate of 10 K min^−1^. The leftover mass of the sample equals the ash content, as all the compounds were oxidized.

In Fourier transform infrared spectroscopy (FT‐IR), the spectra of the outer surface of the balls and the precipitates were acquired using a Bruker ALPHA II PLATINUM‐ATR and are determined by the average of 24 scans for wavenumbers between 4000 and 400 cm^−1^.

In Scanning electron microscopy (SEM), the surface was analyzed concerning the morphology of the hydrothermally treated balls and precipitated material by using a *FEI* Inspect F at the Queen Mary University in London. The dust, scratched from the outer surface of the balls, as well as ground parts of the precipitate were fixed on a carbon tab, respectively, and sputtered with gold for 90 s.

Sample preparation for the liquid analysis was done according to Pfersich et al.^[^ ^25^, ^58^ ^]^ The Ultra Performance Liquid Chromatography (UPLC) with an UPLC gradient device of *Waters Acquity* as well as the Ion chromatography with both, an Integrion‐RFIC and ICS 5000, of *Thermo Scientific*.

The Gas chromatography with Mass spectrometry (GC‐MS) with Gas chromatograph 7890 B and Mass spectrometer 7000 D, both of *Agilent Technologies*, were performed by the Core Facility of the University of Hohenheim. For each sample, the following pretreatment was performed. 150 µL of the clear supernatant after homogenization and centrifugation were dried, 250 µL ethanol added and dissolved in the supersonic bath. After centrifugation (15 min), the supernatant was separated. 250 µL acetone was added to the remaining liquid, dissolved in the supersonic bath for 5 min and, after 15 min centrifugation, the supernatant was added to the first supernatant. The vessel with the mixed supernatants was dried and the resulting supernatant derivatized with 50 µL pyridine and 200 µL BSTFA (30 min at 60 °C). Samples were filled in a vessel and immediately analyzed. The GC column used in this study was an Agilent HP‐5MS UI, 30 m × 250 µm × 0.25 µm, injection mode pulsed splitless, injector temperature 300 °C; column temperature program: 2 min at 85 °C, increasing by a rate of 5 °C min^−1^ to 185 °C, afterward increasing the temperature by a rate of 25 °C min^−1^ to 315 °C and keeping this temperature for 5 min. Helium with a constant flow of 1 mL min^−1^ was used as carrier gas during the analysis.

Mass spectra were recorded in electron impact (EI) ionization mode at 70 eV. The quadrupole mass detector, ion source and transfer line temperatures were set at 150, 200, and 250 °C, respectively. Mass spectrometry was performed in SIM (single ion monitoring) mode (target compounds see **Table** **2**). Identification of volatile compounds was determined by comparing their mass spectra with the data system library Mass Hunter Workstation Software (Agilent) and linear retention index.

**Table 2:** Target compounds of the GC/MS spectrometry with respective retention time on the column and SIM Quantifier and Qualifier masses.

No.	Compound name	Retention time [min]	Quantifier [*m*/*z*]	Qualifier [*m*/*z*]
1	4‐Hydroxybenzaldehyde	13.16	179	151, 194
2	Guaiacol	9.60	181	166, 196
3	Vanillin	17.15	194	209, 224
4	Syringol	13.91	196	211, 226
5	2‐Naphthol (ISTD)	17.75	201	145, 26
6	Guaiacyl acetone	19.59	209	179, 252
7	Homo vanillic acid	22.53	209	311, 326
8	Syringaldehyde	20.97	224	239, 254
9	Acetosyringone	22.45	238	253, 268
10	Orcinol	15.15	253	119, 268
11	Catechol	11.90	254	73, 239

Determination of the dissolved organic carbon (DOC) and total organic carbon (TOC) were done according to Arauzo et al.^[^ ^90^ ^]^

## Conflict of Interest

The authors declare no conflict of interest.

## Supporting information

Supporting Information

## Data Availability

Research data are not shared.
