# Supplementary material for: Evaluation of the Char Formation During the Hydrothermal Treatment of Wooden Balls
Source: Glob Chall. 2023 Nov 5;7(12):2300169. doi: 10.1002/gch2.202300169 (PMC10714026; doi:10.1002/gch2.202300169)
Supplement: Supplementary file 1 — Supporting Information [file GCH2-7-2300169-s001.pdf]

Global Challenges

Open Access

Supporting Information

for *Global Challenges.*, DOI 10.1002/gch2.202300169

Evaluation of the Char Formation During the Hydrothermal Treatment of Wooden Balls

Jens Pfersich, Pablo J. Arauzo, Pierpaolo Modugno, Maria-Magdalene Titirici and Andrea Kruse*

Supporting Information

Evaluation of the char formation during the hydrothermal treatment of wooden balls.

Jens Pfersich, Pablo J. Arauzo, Pierpaolo Modugno, Maria-Magdalene Titirici, Andrea Kruse*

Abstract

With wooden balls, a visualization of the hydrothermal carbonization to show the progress of the conversion to char is presented. In the present study, the balls represent the particles of biomass to investigate the differences in conversion outside and inside of biomass particles, during hydrothermal carbonization. A special focus is on hydrochar and pyrochar formation. The wooden balls were treated in subcritical water at 220 °C for holding times between 0 min and 960 min. Even after 960 min, hydrolysis of the original biomass was incomplete as cellulose and hemicellulose are linked by lignin, inhibiting the reaction with water. Moreover, two different pathways of char production could be observed. Inside of the wooden ball pyrochar was formed as any water got that deep in, on the surface hydrochar was fixed, originated from the surrounding liquid. On the ground of the HTC reactor, a thin, brittle precipitate of likely hydrochar or humins could be found either from the precipitation of loosely attached compounds on the surface of the biomass or direct precipitation from the liquid.

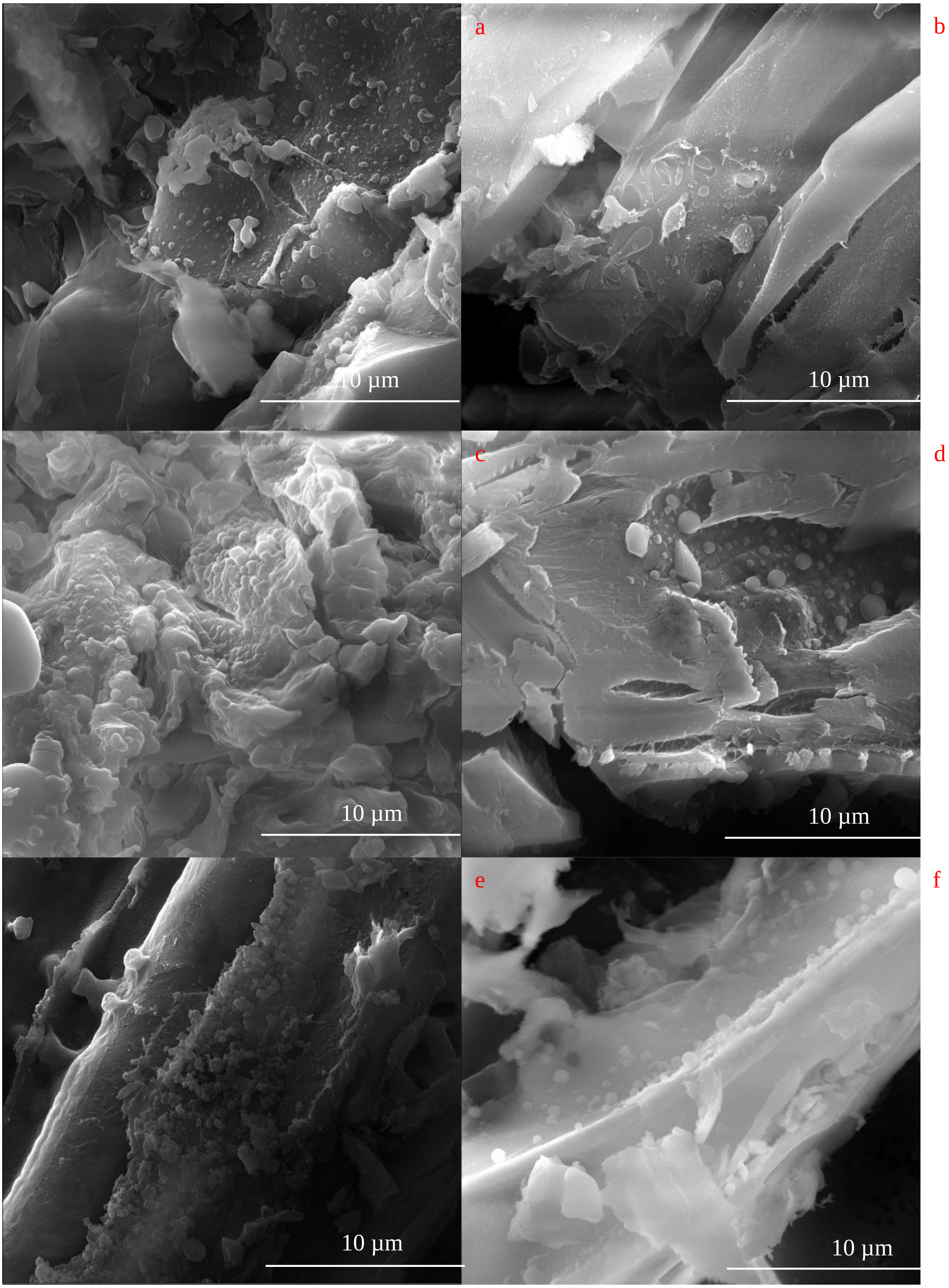

Figure S1. a) SEM image (HV 20.00 kV, mag 10 000x, WD 11.4 mm) of the outer layer of the WB after heating the reactor to the desired reaction temperature for HTC (0 min). b) SEM

image (20.00 kV, mag 10 000x, WD 12.3 mm) of the outer layer of the WB-30-NW after 30 min of HTC. c) SEM image (HV 20.00 kV, mag 10 000x, WD 11.2 mm) of the outer layer of the WB-30-W after 30 min of HTC. d) SEM image (HV 20.00 kV, mag 10 000x, WD 12.0 mm) of the outer layer of the WB-60-W after 60 min of HTC. e): SEM image (HV 20.00 kV, mag 10 000x, WD 11.5 mm) of the outer layer of the WB-120-W after 120 min of HTC. f) SEM image (HV 20.00 kV, mag 10 000x, WD 12.3 mm) of the outer layer of the WB-240-W after 240 min of HTC.

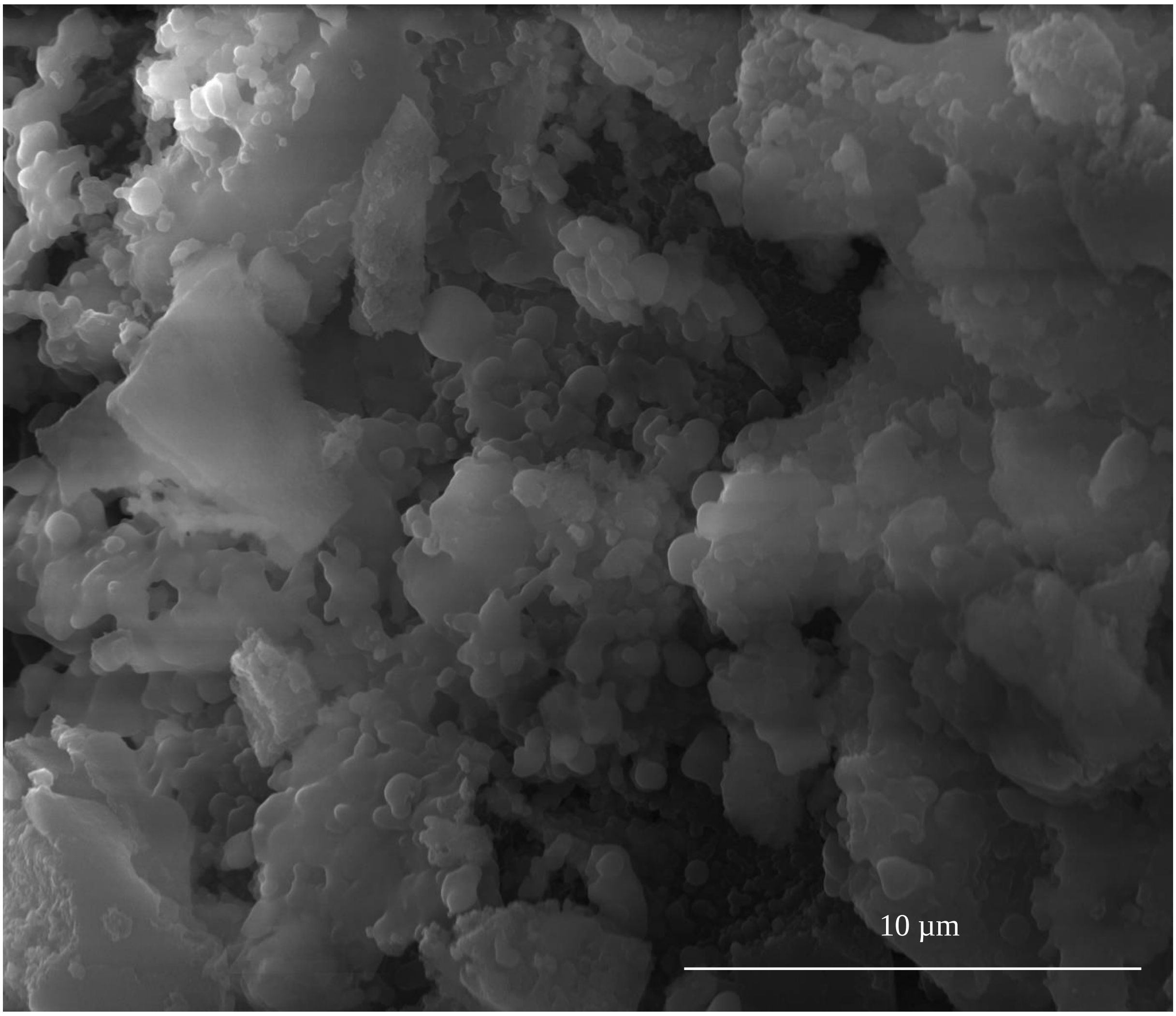

Figure S2. SEM image (HV 20.00 kV, mag 10 000x, WD 12.3mm) of the outer layer of the WB-480-NW after 480 min of HTC.

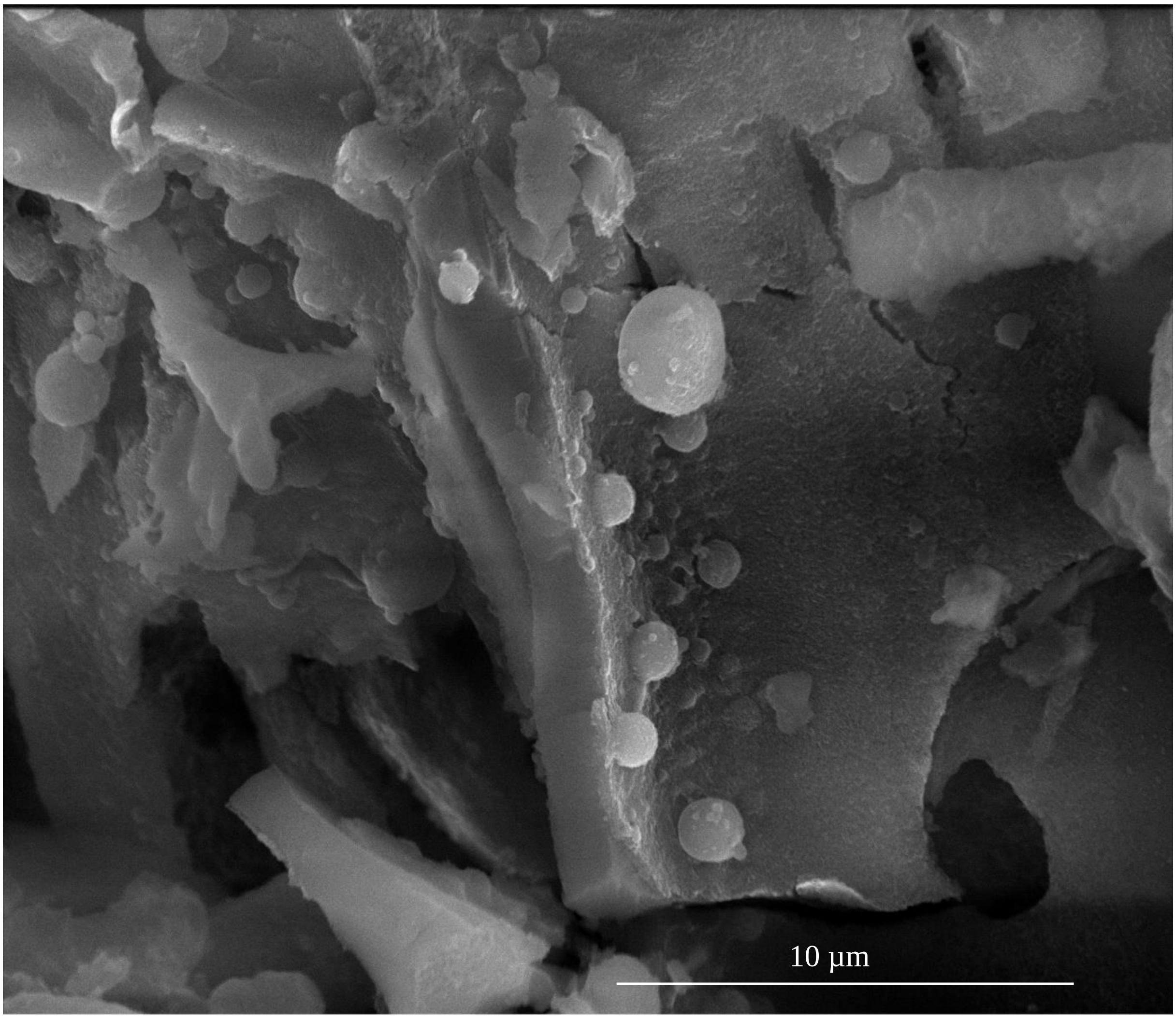

Figure S3. SEM image (HV 20.00 kV, mag 10 000x, WD 12.3 mm) of the outer layer of the WB-480-W after 480 min of HTC.

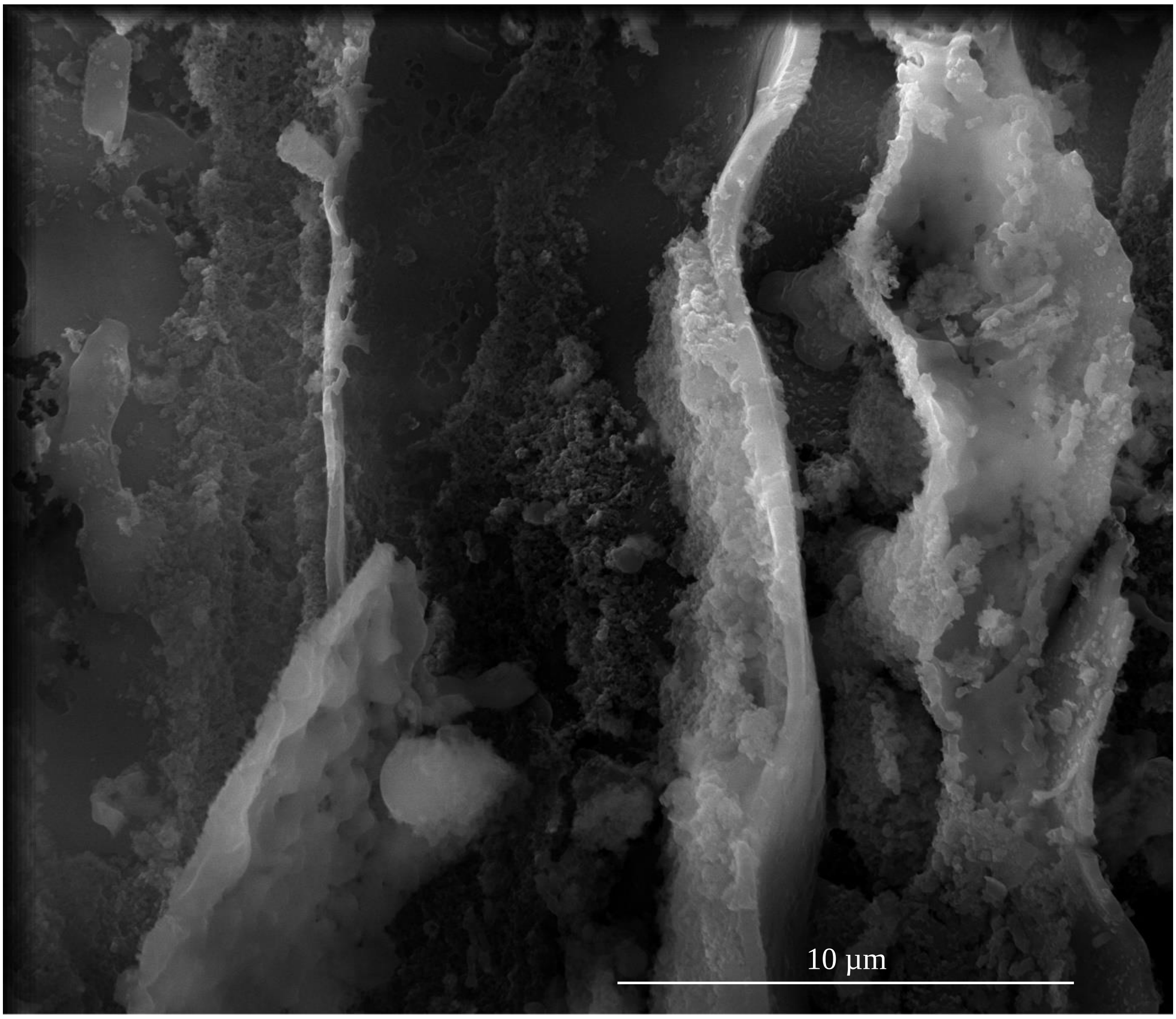

Figure S4. SEM image (HV 20.00 kV, mag 10 000x, WD 11.7 mm) of the outer layer of the WB-960-W after 960 min of HTC.

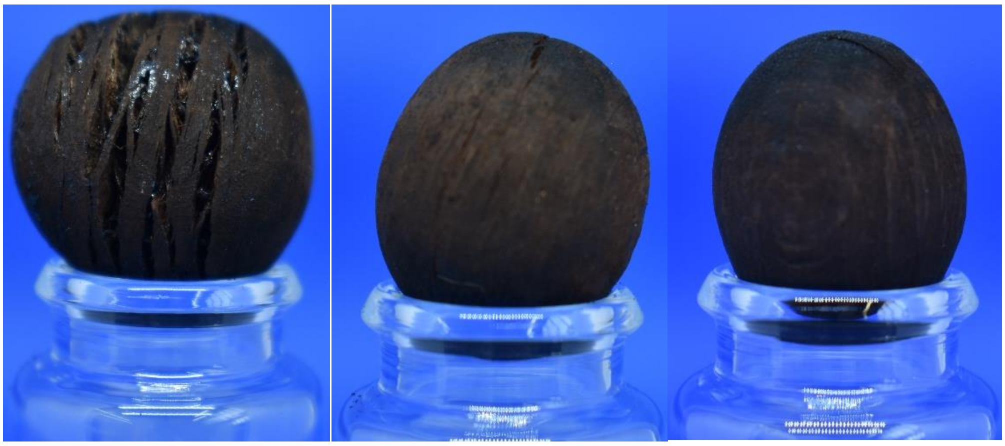

Figure S5. Cracks in the WB after HTC for different times.

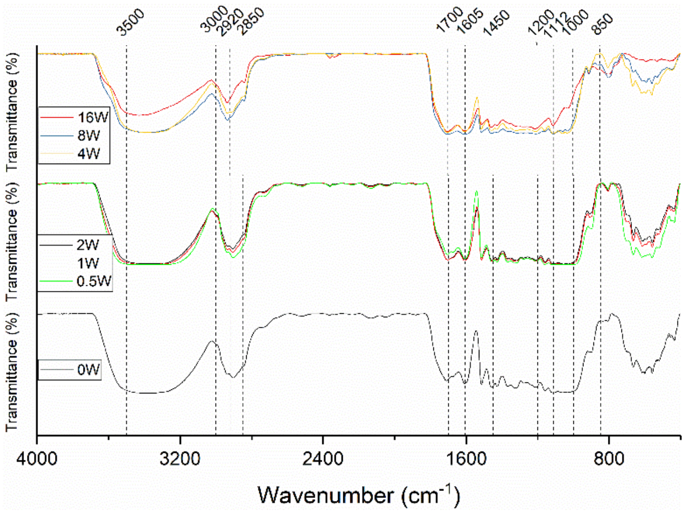

Figure S6. ATR-FTIR spectra of the surface of the wooden balls (washed) after retention times of 0 min to 960 min of HTC at 220 °C.

Table S1. Swelling of WBs by means of water uptake of 6 WBs within 4 h at room temperature.

Swelling time [min]	Mass of water uptake [g]	Rate of swelling [%]
0	0	0
60	2.78 +/- 0.01	12.68 +/- 0.12
120	3.90 +/- 0.02	17.78 +/- 0.13
180	4.52 +/- 0.02	20.61 +/- 0.13
240	4.97 +/- 0.02	22.66 +/- 0.13

Table S2. Yield of hydrochar.

	Yield of Hydrochar [%]
WB220_0	63.1 ± 0.3
WB220_30	60.4 ± 0.4
WB220_60	56.5
WB220_120	51.4 ± 0.7
WB220_240	45.6 ± 0.6
WB220_480	39.7
WB220_960	39.2

Table S3. Results (sugars, furfural and HMF) of the HPLC of the liquid samples after HTC.

	Glucose [mg l^{-1}]	Fructose [mg l^{-1}]	Furfural [mg l^{-1}]	HMF [mg l^{-1}]
WB220_0	242	67	7650	1020
WB220_0	322	82	6680	1080
WB220_30	167	37	5220	2020
WB220_30	196	45	4550	1720
WB220_60	113	26	4180	2090
WB220_60	254	47	4710	2240
WB220_120	197	40	2730	2390
WB220_120	211	45	2750	2490

WB220_240	103	21	930	1850
WB220_240	142	26	1150	1960
WB220_480	34	8	160	510
WB220_960	2	n.n.	14	22

Table S4. Results (acetate, formiate, propionate and levulinic acid) of the HPLC of the liquid samples after HTC.

	Acetate [mg l^{-1}]	Formiate [mgl^{-1}]	Propionate [mg l^{-1}]	Levulinic acid [mg l^{-1}]
WB220_0	8430	870	112	38
WB220_0	7440	881	109	51
WB220_30	9520	1120	158	99
WB220_30	8490	896	139	89
WB220_60	9830	1010	181	138
WB220_60	9040	934	141	138
WB220_120	9600	982	177	236
WB220_120	8330	945	174	225
WB220_240	9990	835	198	343
WB220_240	10430	924	173	388
WB220_480	10230	474	182	647
WB220_960	10610	181	177	778

Equation S1. Calculation of the degree of welling of the wooden balls in contact with water, where m(biomass) is the mass of the original biomass and m(water absorbed) is the mass of the water taken up by the biomass.

$$\text{degree of swelling [\%]} = \left(\frac{\text{m (biomass)} + \text{m (water absorbed)}}{\text{m (biomass)}} - 1\right) \cdot 100\ \% \qquad [1.1]$$
